# Leuven-Haifa High-Resolution Fundus Image Dataset for Retinal Blood Vessel Segmentation and Glaucoma Diagnosis

**DOI:** 10.1038/s41597-024-03086-6

**Published:** 2024-02-29

**Authors:** Jan Van Eijgen, Jonathan Fhima, Marie-Isaline Billen Moulin-Romsée, Joachim A. Behar, Eirini Christinaki, Ingeborg Stalmans

**Affiliations:** 1 Research Group of Ophthalmology, Department of Neurosciences, KU Leuven, Oude Markt 13, 3000 Leuven, Belgium; 2 Department of Ophthalmology, University Hospitals UZ Leuven, Herestraat 49, 3000 Leuven, Belgium; 3 Faculty of Biomedical Engineering, Technion-IIT, Haifa, Israel; 4 Department of Applied Mathematics Technion-IIT, Haifa, Israel

**Keywords:** Diagnostic markers, Neurodegeneration, Atherosclerosis

## Abstract

The Leuven-Haifa dataset contains 240 disc-centered fundus images of 224 unique patients (75 patients with normal tension glaucoma, 63 patients with high tension glaucoma, 30 patients with other eye diseases and 56 healthy controls) from the University Hospitals of Leuven. The arterioles and venules of these images were both annotated by master students in medicine and corrected by a senior annotator. All senior segmentation corrections are provided as well as the junior segmentations of the test set. An open-source toolbox for the parametrization of segmentations was developed. Diagnosis, age, sex, vascular parameters as well as a quality score are provided as metadata. Potential reuse is envisioned as the development or external validation of blood vessels segmentation algorithms or study of the vasculature in glaucoma and the development of glaucoma diagnosis algorithms. The dataset is available on the *KU Leuven Research Data Repository* (RDR).

## Background & Summary

The retinal microvasculature is the only vascular structure in the body that can directly be seen, imaged, and therefore quantified with high resolution. A retinal fundus camera acts as a low-power microscope and acquires red-green-blue images of the posterior pole of the eye including retinal tissue, retinal vessels and the optic nerve head. Eye diseases as glaucoma, microvascular diseases as chronic kidney disease and heart failure and the risk of future cardiovascular events are increasingly correlated to changes in the retinal vessel tree^1–5^. However, in order to enable state-of-the-art parametrization of these vessels, fundus images are often manually segmented, which can take up to several hours per image.

Previously, work has been done on automated blood vessel segmentation based on fundus images. These algorithms achieve dice scores ranging from 80–89, often do not distinguish arterioles from venules, are rather small in size (n = 20 to 40 fundus images) and sometimes lack basic metadata as age or sex. Some algorithms that do report on arteriole/venule segmentation report high and biased accuracy metrics e.g. by taking the weighted average of the arteriole/venule performances and the far more predominant background performance (distinguishing background pixels)^6–17^.

A larger dataset containing fundus images and their associated, manually annotated segmentations and clinical metadata, would allow for the development of accurate and fully automated arteriole/venule segmentation algorithms, the consequent computation of microvascular biomarkers, quantitative analysis thereof and the development of diagnostic or monitoring algorithms.

## Methods

Human data were obtained within the context of the study “Automatic glaucoma detection, a retrospective database analysis” (study number S60649) which consists of 115,668 optic disc-centered fundus retinal images from 13,249 unique patients who visited the University Hospitals Leuven’s glaucoma clinic between 2010 and 2019. The Ethics Committee Research UZ/KU Leuven approved this study in November 2017 and waived the need for informed consent given its retrospective design and because the link between patient ID and study ID was removed upon data export. The research followed the tenets of the Declaration of Helsinki.

From S60649 the *Leuven-Haifa High-Resolution Fundus Image Dataset* was extracted which includes 240 digital fundus retinal images from 224 unique patients (both eyes in n = 16) with normal tension glaucoma (n = 75), high tension glaucoma (n = 63), patients with other eye disease (n = 30) and healthy participants (n = 56). After a first random sampling stratified according to sex and eye laterality, active learning was used to proactively select DFIs in which a preliminary version of the LUNet algorithm had low performance (high lack of vessel continuity)^18^.

The patients included in the dataset are between 18 and 90 years old with a median age of 62 (Q1 and Q3 were 50 and 73 years old) and 42% are male. The dataset contains 57% left eye images. The images were taken with a non-mydriatic fundus camera (*Visucam* 500; *Carl Zeiss Meditec*, Jena, Germany) at an angle of 30 degrees field of view and centered around the optic disc. The resolution of these images is 1444 × 1444 pixels, which results in a high-resolution fundus (HRF) image database that enables the visualization of smaller blood vessels. Low-quality images as indicated by the quality algorithm *FundusQ-Net* (scores lower than 6) were not included in the dataset. *FundusQ-Net* is a deep learning regression model that can estimate fundus image quality relative to a new open-access quality scale (range 1–10). Details on this novel quality scale and the assessment algorithm are published elsewhere^19^.

The blood vessels of these 240 digital fundus images were manually annotated by 15 junior annotators (master students in medicine) and subsequently corrected by a senior annotator (medical doctor, resident in ophthalmology and PhD student with focus on retinal biomarkers for more than 5 years). The resulting segmentations were parametrized by an existing open-source toolbox, the *Python Vasculature BioMarker toolbox* (*PVBM toolbox*)^20^. The *PVBM toolbox* is a fully automated computerized toolbox that enables the computation of vasculature biomarkers engineered from segmented arteriolar or venular networks.

Segmentations were executed between July 2021 and October 2022 on an *Apple iPad Pro 11”* or *13”* using the application *Lirot.ai* which is a platform for facilitating and crowd-sourcing image segmentations. The *Lirot.ai* consists of a python API named *Lirot.ai-API*, a firebase server named *Lirot.ai-server*, and an iPadOS application called *Lirot.ai-app*. With this platform, the researcher initially decides what images need to be segmented and then uses the python Lirot.ai-API to transfer these images to the Lirot.ai-server. The annotators are notified through the Lirot.ai-app and can download the images, segment them and upload the segmentations to the Lirot.ai-server. Finally, the researcher can download the segmentations from all annotators and use them, for example, to train a DL segmentation model. *Lirot.ai* is described in detail elsewhere^21^. In our dataset, arterioles and venules were annotated separately with the *Apple Pencil 2*. A protocol for arteriole/venule discrimination was provided to all annotators and included the following rules: (1) arterioles are redder and venules are darker/more purple; (2) a brighter inner part of a certain vessel, named the central reflex, is typical for arterioles; (3) vessels of the same class very rarely cross meaning that it is unlikely that arterioles cross arterioles or that venules cross venules; (4) venules and arterioles usually alternate near the optic disc.

The 12 parameters that were calculated for arterioles and venules separately using the *PVBM toolbox* are listed below. Detailed description of the *PVBM toolbox* and the calculation of these parameters can be found elsewhere^20^.

1. area (OVAREA)
2. length (OVLEN)
3. perimeter (OVPER)
4. median branching angle (BA)
5. number of endpoints (END)
6. number of intersection points (INTER)
7. median tortuosity (TOR)
8. tortuosity index (TI)
9. capacity dimension (D0)
10. entropy dimension (D1)
11. correlation dimension (D2)
12. singularity length (SL)

**Fig. 1:**
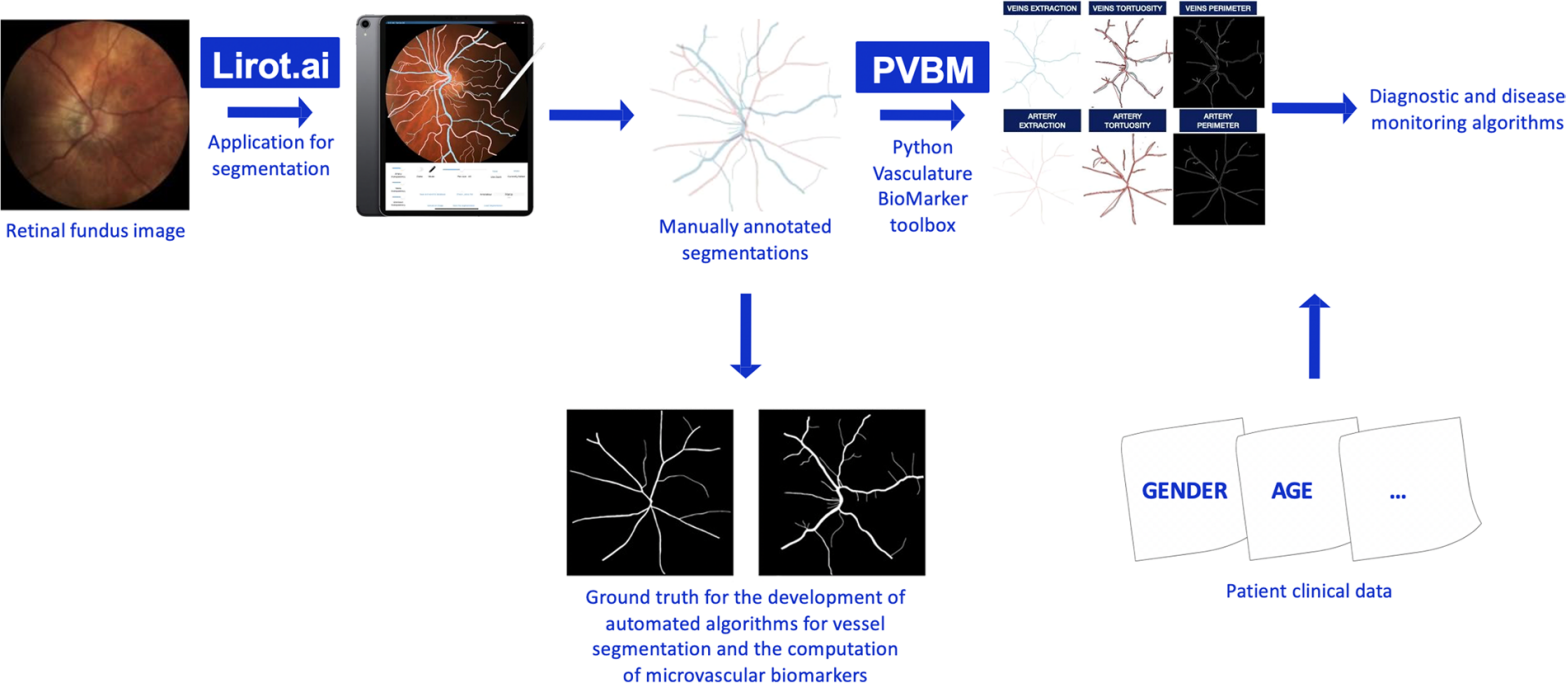
Segmentation pipeline and suggestions for algorithm development. Raw fundus images are manually annotated in the Lirot.ai application by junior annotators and corrected by an expert annotator^21^. The resulting manual segmentations could serve as ground truth for vessel segmentation algorithm development and could be used for the computation of microvascular biomarkers, e.g. *PVBM toolbox*^20^. Together with clinical metadata these biomarkers carry the potential for disease screening and risk stratification, classification and monitoring algorithms.

The fundus images with their respective segmentations (arterioles and venules both combined and separately), clinical metadata (age, sex, diagnosis, eye laterality), image quality score (*FundusQ-Net*), calculated parameters and *PVBM Toolbox* code are assembled in this Leuven-Haifa dataset. An overview of this processing pipeline can be found in Fig. 1.

## Data Records

The data records are reposited in the RDR^22^, are protected under Data Usage Agreement, and can be downloaded after completing a request form as is stipulated in the study protocol due to local regulations.

**Fig. 2:**
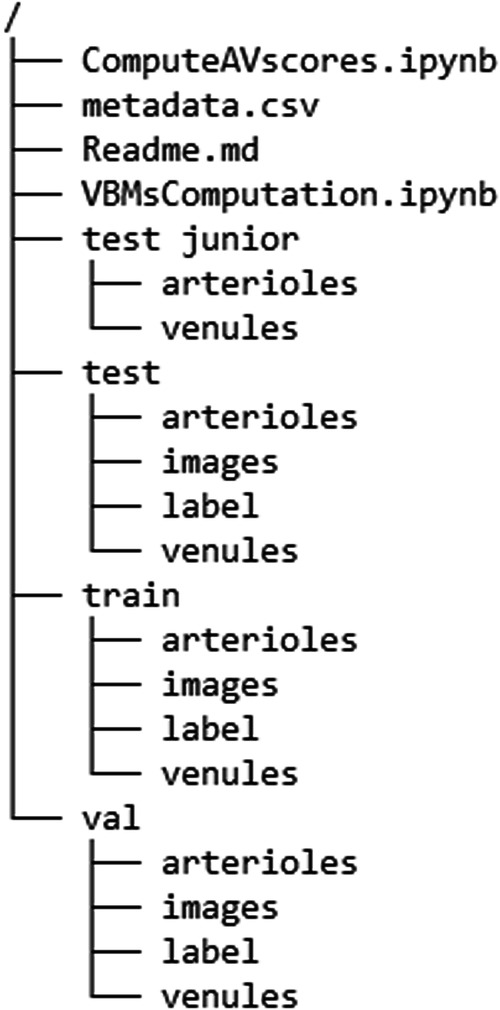
Data records root structure. Detailed information on the root directory can be found in the section *Data Records*.

At the root, there are three files named metadata.csv, VBMsComputation.ipynb and ComputeAVscores.ipynb and four directories named train, val, test and test junior (Fig. 2).

metadata.csv is a CSV file that holds the following information: image file name, image id, eye, patient id, day of fundus image (dayDFI), gender, age, diagnosis, quality score and vasculature parameters corresponding to each image present in the dataset. Of note: dayDFI represents the number of days an image was taken after a certain point in the past.

Diagnosis contains the following four classes:

- Normal - patients with normal ophthalmic findings
- NTG - patients with normal tension glaucoma
- POAG - patients primary open angle glaucoma
- Other - patients with another eye disease

VBMsComputation.ipynb is a notebook file which contains the code used to compute the vasculature biomarkers using the *PVBM toolbox*.

ComputeAVscores.ipynb is a notebook file with the code used to calculate the artiole/venule dice scores of the junior test set compared to the senior test set.

The four directories, namely train, val, test and test junior, host training, validation, and testing data of the senior annotator and the testing data of the junior annotator respectively. The image set has been divided at the patient level into the training set (n = 156), the validation set (n = 56) and the test set (n = 28) at 3:2:1 ratio, randomly sampling in each diagnosis class, balancing sex and eye laterality, making sure that multiple pictures of the same patient are all allocated to the test set. Each of these directories further contains four folders: arterioles, venules, label, and images which contain the following files:

- The arterioles and venules folder are the segmentations representing arterioles and venules separately (.png).
- The label folder holds composite segmentations (.png). The red color signifies arterioles, the blue color indicates venules.
- The images folder contains all raw fundus images (.png).

**Fig. 3:**
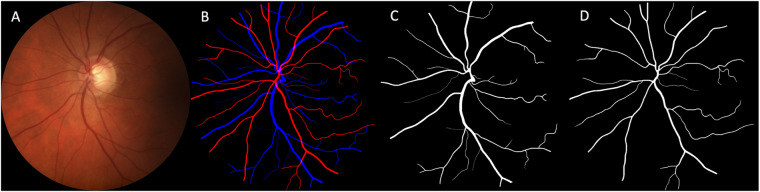
Example of a single.png entry of the Leuven-Haifa dataset. (**A**) A raw retinal image of the left eye. (**B**) A composite segmentation containing overlayed arteriolar and venular segmentations. (**C**) Segmentation of the corresponding venules. (**D**) Segmentation of the corresponding arterioles.

Files that correspond to a certain fundus image are named identically in every folder. The file naming, which can be also found in the metadata csv file, is structured as follow: “ImageID_Eye_PatientID_Day_Gender_Age” (e.g., 001323_LR_90852_03348_M_46). Of note, in the file naming the notation LX corresponds to the left eye while RX corresponds to the right eye. A representative example of a raw fundus image and corresponding segmentations can be found in Fig. 3.

## Technical Validation

Low-quality images were excluded from the dataset (*FundusQ-Net* < 6)^19^.

The code in ComputeAVscores.ipynb was used to calculate the performance of the junior annotations compared to the senior corrections. The test junior set achieved a dice score of 82% and 84% for arterioles and venules respectively.

As enforced by the FLAIR (Findable, Accessible, Interoperable, and Reusable) and TRUST (Transparency, Responsibility, User focus, Sustainability, and Technology) principles, major challenges within machine learning practice that encompass robustness, trustworthiness and comparability call for multiple labeling and quality metrics as time spent to label and statistics of consistency amongst labelers, classes or datasets^23,24^. For this purpose the authors suggest to future users of this dataset to label the images, to calculate quality metrics thereof (an example of which can be achieved by ComputeAVscores.ipynb) and to make these results publicly available in a linked repository.

Primary open angle glaucoma diagnosis was made following the latest European Glaucoma Society guidelines. A maximal untreated intra-ocular pressure of 21 mmHg and lower classified these patients as normal tension glaucoma. The diagnosis *Other* refers to a.o. papilledema, cataract, central retinal vein occlusion, hypertensive retinopathy, myopia, status after refractive surgery, status after vitrectomy (for vitreous haemorrhage or retinal detachment), status after strabismus surgery, status after surgery for congenital cataract, pigment dispersion syndrome, ocular hypertension, juvenile open-angle glaucoma, closed angle glaucoma, secondary glaucoma and patients that are suspected, yet unconfirmed, for established primary open-angle glaucoma^25^.

## Usage Notes

The whole dataset can be downloaded from the RDR repository at 10.48804/Z7SHGO. Data access must be formally requested via RDR repository as follows:

- Use the Contact Owner button at the top of the dataset page to send the contact person a message to go with your request, providing your mail address and name.
- Specify why you want access to which specific file(s). Make sure to start the subject field with “Access Request:”.

After this initial step a request form will be sent to the mail address of the requester with the following fields and questions:

- Email (must be from an academic institution), First name, Last name, Title, Institution (university, faculty, department or laboratory), Address, Zip code, City, Country, Phone, Website.
- Research goals on the data set.

This information will be used to provide the requester with a Data Usage Agreement (DUA). After signature of the DUA by the requester and Principal Investigator an expirable download link will be sent to the interested user.

Our dataset is meticulously organized to facilitate easy navigation and usage. The images folder stores the original, raw, unlabeled images. This is particularly useful for researchers who may wish to implement their proper labelling system. By structuring our dataset in this manner, we aim to facilitate user-friendly access to an extensive array of images and corresponding data, thereby fostering research in the areas of:

- fully automated arteriole/venule segmentation algorithms
- computation of microvascular biomarkers and quantitative analysis thereof
- diagnostic and disease monitoring algorithms

We recommend researchers to use or validate the *PVBM Toolbox* for the computation of an extensive array of vascular parameters^20^. Secondly, researchers can compare the performance of their derived segmentation algorithm to the author’s deep learning LUNet algorithm trained on this dataset^18^.

## Data Availability

We provide the *PVBM toolbox* code used to extract the microvascular biomarkers within the RDR repository as a notebook file^22^. The PVBM toolbox code is also available via https://pvbm.readthedocs.io/en/latest/. For reproducibility and convenience in case any user wants to customize the extraction, all the .py files and a *Readme* file are available.
